# Vitamin D Inadequacy and Its Relation to Body Fat and Muscle Mass in Adult Women of Childbearing Age

**DOI:** 10.3390/nu16091267

**Published:** 2024-04-25

**Authors:** Paula Moreira Magalhães, Sabrina Pereira da Cruz, Orion Araújo Carneiro, Michelle Teixeira Teixeira, Andréa Ramalho

**Affiliations:** 1 Postgraduate Program of Clinical Medicine, Faculty of Medicine, Federal University of Rio de Janeiro (UFRJ), Rio de Janeiro 21044-020, Brazil; 2 Center for Research on Micronutrients (NPqM), Institute of Nutrition Josué de Castro, Federal University of Rio de Janeiro (UFRJ), Rio de Janeiro 21941-902, Brazil; sabrina.cruz.ufrj@gmail.com (S.P.d.C.); nutricionistaorion@uol.com.br (O.A.C.); aramalho.rj@gmail.com (A.R.); 3 Department of Public Health Nutrition, Nutrition School, Federal University of the State of Rio de Janeiro (UNIRIO), Rio de Janeiro 22290-250, Brazil; michelle.teixeira@unirio.br; 4 Department of Social and Applied Nutrition, Institute of Nutrition Josué de Castro, Federal University of Rio de Janeiro (UFRJ), Rio de Janeiro 21941-902, Brazil

**Keywords:** vitamin D, body composition, women of childbearing age, body fat, muscle mass

## Abstract

To assess the correlation between vitamin D status and body composition variables in adult women of childbearing age, a cross-sectional study was conducted involving women aged 20–49 years. The participants were categorized based on their vitamin D status and further divided according to body mass index (BMI). Anthropometric and biochemical data were collected to compute body composition indices, specifically body fat and muscle mass. The sample included 124 women, with 63.70% exhibiting vitamin D inadequacy. Women with inadequate vitamin D status demonstrated a higher waist-to-height ratio (WHtR) and body adiposity index (BAI), along with a lower BMI-adjusted muscle mass index (SMI _BMI_), compared to those with adequate levels of vitamin D (*p* = 0.021; *p* = 0.019; and *p* = 0.039, respectively). A positive correlation was observed between circulating concentrations of 25(OH)D and SMI _BMI_, while a negative correlation existed between circulating concentrations of 25(OH)D and waist circumference (WC), WHtR, conicity index (CI), fat mass index (FMI), body fat percentage (% BF), and fat-to-muscle ratio (FMR). These findings suggest that inadequate vitamin D status may impact muscle tissue and contribute to higher body adiposity, including visceral adiposity. It is recommended that these variables be incorporated into clinical practice, with a particular emphasis on WHtR and SMI _BMI_, to mitigate potential metabolic consequences associated with vitamin D inadequacy.

## 1. Introduction

The prevalence of vitamin D deficiency (VDD) in women of childbearing age is high [1,2,3,4,5,6], especially in those with obesity (88%) compared to those with a normal weight (51%) [7]. Evaluating the nutritional status of this vitamin is important not only for promoting bone, reproductive, immune, and mental health but also for understanding the influence of vitamin D on different tissues, such as adipose tissue and skeletal muscle, which have specific receptors for this vitamin [8,9,10,11].

In recent years, there has been a growing interest in understanding the relationship between vitamin D and body composition, as evidenced by the association between VDD and obesity [12]. However, most studies rely on conventional measures such as body mass index (BMI), waist circumference (WC), and body fat percentage (% BF) to assess body composition [13,14,15,16,17,18]. These studies often overlook other important variables related to the distribution of body fat.

Similarly, there is evidence of the association of vitamin D with skeletal muscle mass (SMM), where VDD can contribute to the reduction of muscle mass as well as decreased strength and muscle function, leading to sarcopenia [19]. Understanding these aspects is essential for a comprehensive assessment of the influence of vitamin D on body composition and muscle health in women of childbearing age.

Considering the crucial role of this vitamin in muscle mass during pregnancy and postpartum, influencing functional capacity and quality of life, this relationship promotes functional autonomy and reduces the risk of complications associated with muscle health throughout life. However, no studies related to the behavior of these variables in women of childbearing age have been found [20,21].

Considering the limited number of studies addressing the relationship between vitamin D status and body composition variables among adult women of childbearing age, especially in the context of body fat distribution and muscle mass, the present study aims to assess this association. These variables enable the measurement of both body fat and muscle mass in women within this age range.

## 2. Materials and Methods

This is a cross-sectional study with adult women of childbearing age assisted by a nutrition service located in the city of Rio de Janeiro, Brazil. Data collection occurred between August 2018 and August 2019. The participants were required to undergo clinical-nutritional anamnesis along with anthropometric and biochemical evaluation during the study period.

The sample size was calculated based on a prevalence study of VDD and its associated factors in women of childbearing age in Brazil [22]. To achieve a sample size with a 95% confidence interval, considering a prevalence of inadequacy of 72% with a margin of error of 5%, 86 women of childbearing age would be required in order to conduct the present study.

Women aged 20–49 years, of a normal weight and overweight (overweight and obesity) according to BMI and who provided information on the research variables, were included in the study. The exclusion criteria were pregnant women, nursing mothers, menopausal women, women who had undergone bariatric surgery, and those who had received vitamin D supplementation in the last 6 months.

Circulating concentrations of 25-hydroxyvitamin D (25(OH)D) were determined via high-performance liquid chromatography with ultraviolet detector (HPLC-UV) [23]; the reference values of Holick et al. (2011) [24] were adopted, and were evaluated according to circulating concentrations of 25(OH)D as sufficient (25(OH)D ≥ 30 ng/mL), insufficient (25(OH)D ≥ 20 and < 30 ng/mL), and deficient (25(OH)D < 20 ng/mL). In the current study, circulating concentrations of 25(OH)D ≥ 30 ng/mL were considered adequate and circulating concentrations of 25(OH)D < 30 ng/mL inadequate. In addition, triglycerides and high-density lipoprotein (HDL-cholesterol) serum concentrations were determined using a colorimetric enzymatic method, and their values were used to calculate one of the body variables.

Anthropometric evaluation consisted of measuring body weight in kilograms using an InBody270 electronic anthropometric scale [25]; height in meters using an Avanutri^®^ (Avanutri, Três Rios, Brazil) stadiometer [25]; WC, hip circumference (HC) and arm circumference (AC) in centimeters measured with a Sanny^®^ (Sanny, São Bernardo do Campo, Brazil) tape measure [25,26,27]; and triceps skinfold (TSF) in millimeters measured with a Lange^®^ (US Chemical, Greenville, SC, USA) adipometer [25].

With these anthropometric data, the arm muscle circumference (AMC) [28] and the arm muscle area (AMA) [29] were calculated to evaluate muscle mass and body fat, BMI [26], waist-to-hip ratio (WHR) [30], conicity index (CI) [31], waist-to-height ratio (WHtR) [32], visceral adiposity index (VAI) [33], body adiposity index (BAI) [34], a body shape index (ABSI) [35], Clinical Universidad Navarre—body adiposity estimator (CUN-BAE) [36], and Belarmino-Waitzberg index (BeW) [37], according to their published formulas. BMI was assessed according to the (2000) cut-off points for normal weight (BMI ≥ 18.5 and ≤ 24.9 kg/m^2^) and overweight (BMI ≥ 25.0 kg/m^2^) recommended by WHO.

In addition, electric bioimpedance testing was performed with an InBody270^®^ tetrapolar scale. Participants were informed about the pre-examination procedures to reduce measurement errors, obtaining fat mass (FM), fat-free mass (FFM), skeletal muscle mass (SMM) and % BF. With these data, the fat mass index (FMI) [38] and the fat-to-muscle ratio (FMR) [39] were obtained for body fat evaluation. For muscle mass evaluation, the fat-free mass index (FFMI) [38] and the muscle mass indices adjusted by height^2^ (SMI _height_) [40]; body weight (SMI _weight_) [41] and BMI (SMI _BMI_) [42] were considered according to their published formulas.

In Table 1 and Table 2 are shown the overall indices and their formulas used to estimate body fat and muscle mass, respectively.

The study was approved by the Research Ethics Committee of the Federal University of the State of Rio de Janeiro (UNIRIO), and the protocol was registered in the National Research Ethics System (CAEE 50063715.5.0000.5285).

**Figure 1:**
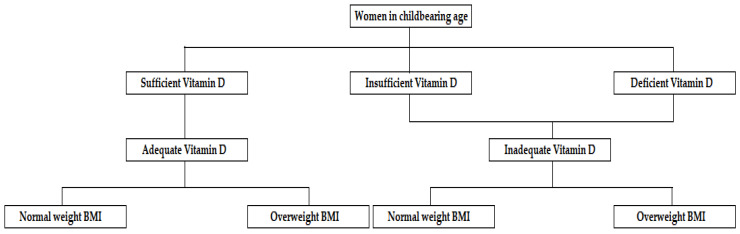
Division of women in the study. Legend: Women were divided according to circulating concentrations of 25(OH)D into sufficient (25(OH)D ≥ 30 ng/mL), insufficient (25(OH)D ≥ 20 and < 30 ng/mL) and deficient (25(OH)D < 20 ng/mL) vitamin D, and into adequate (25(OH)D ≥ 30 ng/mL) and inadequate (25(OH)D < 30 ng/mL) vitamin D. Women with adequate and inadequate vitamin D were divided into normal weight (BMI ≥ 18.5 and ≤ 24.9 kg/m^2^) and overweight (BMI ≥ 25 kg/m^2^) according to BMI.

The women were divided, as illustrated in Figure 1, according to circulating concentrations of 25(OH)D into sufficient, insufficient, and deficient, then into adequate and inadequate, vitamin D. The groups of women with adequate and inadequate vitamin D were then divided, according to BMI, into normal weight or overweight.

Statistical tests were performed in the statistical package SPSS for windows version 21.0. The Kolmogorov–Smirnov test was performed to evaluate the normality of the sample. Measures of central tendency and dispersion (mean and standard deviation) were calculated for quantitative variables, and the Mann–Whitney and Kruskal–Wallis tests were used for comparisons. In comparisons of 2 groups, the Mann–Whitney test was applied, and in comparisons of 3 groups or more, the Kruskal–Wallis test was applied. In the case of comparisons of 3 groups, after the Kruskal–Wallis test, the Mann–Whitney test and Bonferroni correction was applied for separate post-test. The analysis of correlations between serum 25(OH)D concentrations and the analyzed variables was estimated using Spearman’s correlation coefficient. For all statistical tests, a probability of less than 5% (*p* < 0.05) was considered as the statistical significance level. 

## 3. Results

### 3.1. General Characterization of the Sample

A total of 124 women with a mean age of 34.07 ± 7.13 years participated in the study. The mean BMI was 24.20 ± 3.48 kg/m^2^, with 66.93% classified as having a normal BMI, 23.39% as overweight, and 9.68% as obese. The mean of circulating concentrations of 25(OH)D was 28.83 ± 10.35 ng/mL. Of the participants, 36.30% had vitamin D adequacy, while 63.70% had vitamin D inadequacy, including 44.35% with insufficiency and 19.35% with deficiency. 

### 3.2. Body Composition According to Vitamin D Serum Concentrations

The means of 25(OH)D and the variables analyzed based on vitamin D status are presented in Table 3. Upon examining these results, we observed significantly lower BAI values in women with vitamin D sufficiency. 

In the assessment of women with vitamin D inadequacy, significantly higher WHtR and BAI values were found compared to those with adequacy. Additionally, significantly higher SMI _BMI_ values were observed in women with adequacy compared to those with inadequacy. Therefore, participants with some degree of impairment in vitamin D status exhibited higher values in body fat variables, including visceral fat, and lower values in muscle mass, in comparison to those with adequate vitamin D status. 

### 3.3. Body Composition According to Vitamin D Serum Concentrations by BMI

When dividing participants with vitamin D adequacy by BMI, it was observed that 28.23% (n = 35) were of normal weight and 8.06% (n = 10) were overweight. Regarding vitamin D inadequacy, 38.71% (n = 48) were of normal weight and 25% (n = 31) were overweight. 

Concerning the mean body composition variables, significant differences were found in weight, WC, HC, AC, TSF, AMC, AMA, BMI, WHR, WHtR, BeW, BAI, CI, FMI, CUN-BAE, SMM, FM, FFM, % BF, FMR, SMI _height_, SMI _weight_, and SMI _BMI_ among all women assessed (Table 4). It is noteworthy that the WHtR was significantly higher in women of normal weight and vitamin D inadequacy when compared to those of normal weight and vitamin D adequacy. Women who were overweight with vitamin D inadequacy had significantly higher mean values of weight, HC, and BeW than those who were overweight with vitamin D adequacy. 

### 3.4. Correlations of Vitamin D with Body Composition

There was a negative correlation between circulating concentrations of 25(OH)D and WC (r = −0.194, *p* = 0.031), WHtR (r = −0.218, *p* = 0.015), CI (r = −0.207, *p* = 0.021), FMI (r = −0.210, *p* = 0.039), % BF (r = −0.214, *p* = 0.035), FMR (r = −0.226, *p* = 0.026), and a positive correlation with SMI _BMI_ (r = 0.219, *p* = 0.031) (Table 5). 

When only women with vitamin D inadequacy were analyzed, a negative correlation was found between circulating concentrations of 25(OH)D and VAI (r = −0.359, *p* = 0.003). This correlation was higher when only those with deficiency were analyzed (r = −0.520, *p* = 0.022). 

Furthermore, women with insufficiency showed a negative correlation between circulating concentrations of 25(OH)D and % BF (r = −0.336, *p* = 0.028). Those with deficiency, in addition to the negative correlation of 25(OH)D with VAI, also had TSF (r = −0.472, *p* = 0.027). 

## 4. Discussion

In this study, using different body composition assessment methods, a negative correlation of vitamin D status with body fat, especially abdominal fat, and a positive correlation with muscle mass were found. Among the indices showing correlation, the WHtR and SMI _BMI_ stand out and can be easily incorporated into clinical practice.

### 4.1. Prevalence of Vitamin D Inadequacy and Deficiency

The literature highlights a high prevalence of vitamin D inadequacy and deficiency in adult women of childbearing age, similar to what was found in the current study [2,5,22,43,44,45]. Variations in prevalence values may be attributed to different geographical locations of the studied populations, as well as seasonal changes and sun exposure.

A noteworthy recent meta-analysis that involved 4006 participants aimed to assess vitamin D deficiency in women of childbearing age in Brazil. This analysis revealed an overall prevalence of deficiency (35%, 95% CI: 34–37%), insufficiency (42%, 95% CI: 41–44%), and combined deficiency and insufficiency of vitamin D (72%, 95% CI: 71–74%) [22], similar to the findings in the present study.

### 4.2. Relationship between Serum Vitamin D Concentrations and Muscle Mass

Women with vitamin D adequacy exhibited higher muscle mass (SMI _BMI_), and this was positively correlated with the vitamin. Recently, SMI _BMI_ was identified as the most effective adjustment method for SMM, demonstrating a stronger association with functional and disability measures (handgrip strength, gait speed, frailty) than adjustment based on height^2^ and weight [46].

These findings provide important information for clinical assessment, as they suggest a possible influence of vitamin D on muscle tissue. Thus, maintaining sufficient concentrations of vitamin D may possibly ensure better muscle capacity and functionality for these women.

In agreement, other authors have also found a relationship between vitamin D status and muscle tissue. A study involving young (25 to 45 years) and middle-aged (46 to 60 years) adult women found positive correlations between 25(OH)D concentrations and muscle strength (1 repetition maximum in supine and leg press and handgrip) in both age groups assessed [47]. Another study in subjects with BMI ≥ 25 kg/m^2^ of both sexes (60% women) observed that those with vitamin D sufficiency (25(OH)D > 30 ng/mL) had significantly higher SMI _weight_ (% SMM) (31.8 ± 4.9 vs. 36.7 ± 6.1, *p* < 0.001). Moreover, serum vitamin D concentrations were positively associated with SMM (r = 0.18, *p* = 0.03) and maintained their levels after adjustment for fat mass and age (*p* = 0.003) [48].

This relationship can be explained by the presence of the VDR receptor in muscle tissue acting in two ways, as a nuclear receptor mediating genomic effects and through non-nuclear receptors mediating non-genomic actions. Thus, 1,25 dihydroxyvitamin D (1,25(OH)_2_D) may play a role in muscle contraction by increasing calcium mobilization through enhancing the activities of calbindin D9K, a calcium-binding protein, in cellular sarcoplasm [49]. It also activates important molecules of the store-operated calcium entry (SOCE) and voltage-dependent calcium channel (VDCC) pathways, resulting in extracellular calcium influx [50].

Furthermore, 1,25(OH)_2_D can function in cell proliferation, differentiation, and growth through the activation of different molecules present in cell signaling pathways, such as phosphoinositide 3-kinase (PI3K), mitogen-activated protein kinase (MAPK), protein kinase B (AKT), and mammalian target of rapamycin (mTOR) [51]. In addition to increasing the expression of growth factors, such as insulin-like growth factor 1 (IGF-1), insulin-like growth factor 2 (IGF-2), vascular endothelial growth factor A (VEGFa), and fibroblast growth factor 1 (FGF-1) [52,53,54]. Thus, vitamin D may play an important role in increasing muscle mass.

Finally, 1,25(OH)_2_D can also increase mitochondrial oxygen consumption, the volume fraction of mitochondria, and mitochondrial branching [55], as well as exert an inhibitory effect on oxidative stress and mitochondrial dynamics by activating AMPK and sirtulin 1 (SIRT1) in muscle cells [56].

As a result, deficiency of the active form of vitamin D in skeletal muscle can lead to alterations in metabolic pathways, ultimately resulting in decreased protein synthesis, muscle atrophy, and mitochondrial dysfunction [57]. Therefore, it is important to maintain sufficient vitamin D status to ensure its functionality in skeletal muscle.

### 4.3. Relationship between Serum Vitamin D Concentrations and Body Fat

Women with vitamin D inadequacy exhibited higher percent body fat (%BF) and central obesity (WHtR). Additionally, vitamin D was negatively correlated with body fat (WC, WHtR, CI, FMI, %BF and FMR). These findings underscore the importance of assessing body fat through complementary methods that do not rely solely on body weight, as most formulas for these variables use WC or fat mass measured by bioimpedance. Furthermore, these results reinforce that abdominal fat, often more visceral, may be related to vitamin D.

Similarly, Shantavasinkul et al. (2015) observed that individuals with vitamin D adequacy had significantly lower % BF and this was negatively associated with 25(OH)D concentrations [48]. Another study, on the other hand, involving both sexes (40.5% women), found an association of 25(OH)D with % BF, even after adjusting for age and sex, and a negative correlation of 25(OH)D with % BF as well [58]. Nikolova et al. (2018) demonstrated that 25(OH)D was negatively correlated with WC, WHtR, and % BF in women aged 20–59 years [59].

In a study by Patriota et al. (2022) evaluating men and women aged 35–75 years, a negative correlation of 25(OH)D was observed with all anthropometric markers (weight, BMI, WC, HC, WHtR, WHtR, % BF, CI, and body roundness index (BRI)) except ABSI in women. In addition, % CG, WHtR, and BMI were negatively associated with 25(OH)D in women [60]. Another study, by Arazi et al. (2019), found a negative association of 25(OH)D with BMI in both young and adult women. These findings of Patriota et al. (2022) and Arazi et al. (2019) related to BMI, unlike what was found in our study, may have occurred because the overall BMI means of their samples corresponded to overweight BMI (25.1 ± 4.8 kg/m^2^ and 26.91 ± 4.59 kg/m^2^, respectively), which did not occur in our study [47,60].

In women with inadequacy and deficiency of vitamin D, it correlated negatively with visceral fat (VAI). This index aims to assess the distribution and function of visceral fat, as it correlates with factors that make up metabolic syndrome and cardiovascular and cerebrovascular events [33]. Some studies indicate that visceral fat, as measured by the omental compartment, has higher vitamin D concentrations compared to subcutaneous fat [61,62]. Thus, visceral adipose may play a crucial role in the deficiency of this vitamin.

Given the above, it is assumed that vitamin D inadequacy shows a stronger relationship with visceral adiposity, which, in turn, is associated with an increased rate of macrophage infiltration and increased release of pro-inflammatory adipokines that directly enter the portal circulation, promoting inflammation [63]. In addition, the amount of adiponectin is lower in visceral adipose tissue, further contributing to this pro-inflammatory scenario [64]. These alterations may pose significant cardiometabolic risks to these women, particularly hypertension, resistance to insulin action, type 2 diabetes mellitus, dyslipidemia, and cardiovascular diseases [65,66,67,68,69,70].

Furthermore, inadequate vitamin D status seem to be related to the way body fat is distributed, depending on BMI. In normal-weight women, a greater relationship with central obesity (WHtR) stands out; in women with obesity, the relationships with weight, HC, and body fat percentage (BeW) stand out.

All these findings indicate a relationship between vitamin D and adiposity, but caution should be exercised when interpreting these data as they can go both ways. Excess adipose tissue can lead to lower vitamin D concentrations; conversely, lower vitamin D concentrations can result in excess body fat.

In this context, the literature provides some possible explanations for such findings. The first would be the fact that adipose tissue acts as the main reservoir of vitamin D in the body, thus decreasing its bioavailability, in addition to generating the inability of this vitamin to return to the circulation as a substrate for 25-hydroxylase in the liver [71,72,73]. Volumetric dilution is noted as another possible cause of low vitamin D concentrations in individuals with obesity, as vitamin D is distributed in a greater volume of serum, muscle, fat, and liver (increased body compartments in this situation), requiring a greater amount of vitamin D to saturate these deposits, thus decreasing its serum concentrations [61,74,75].

More recently, a study provided evidence to support the existence of an additional mechanism for these findings. Specifically, levels of CYP2R1, the major hepatic 25-hydroxylase, were significantly reduced in mice with obesity. Thus, less 25(OH)D would be converted at the hepatic level, thus explaining lower plasma concentrations [76].

In addition, there are some studies that support the hypothesis that vitamin D may be involved in the pathogenesis of obesity, rather than just a consequence. 1,25(OH)2D plays a role in controlling adipogenesis, energy metabolism, adipokines expression and inflammation, and apoptosis of adipose cells. In mouse preadipocyte cells, 1,25(OH)2D has been shown to stimulate adipogenesis by decreasing the expression of regulatory proteins, leading to terminal differentiation of preadipocytes and other related molecules. However, in human cells there appears to be increased adipocyte differentiation and lipid accumulation in the presence of 1,25(OH)2D through increased expression of adipogenic markers. Therefore, its effects on adipogenesis are still uncertain [77].

Regarding energy metabolism, mice with targeted expression of human VDR in adipocytes develop obesity, due to reduced energy expenditure [78]. In the same animal model, overexpression of VDR in adipose tissue induced increased body weight and fat mass, in addition to a decline in energy metabolism and a state of resistance to insulin action, resulting from a decrease in the expression of molecules important for energy expenditure [10]. In human adipocytes, 1,25(OH)2D inhibited lipolysis and promoted lipogenesis by increasing the expression of adipogenic proteins [79].

Regarding inflammation and adipokine expression, low concentrations of vitamin D were associated with increased inflammatory markers and generation of reactive oxygen species in visceral adipose tissue samples from obese individuals. Furthermore, incubation with 1,25(OH)2D decreased oxidative stress in this tissue [80]. Treatment with 1,25(OH)2D reduced the production of different pro-inflammatory adipokines and attenuated the inflammatory response in adipose tissue through inhibition of signaling pathways, such as nuclear factor kappa B (NFκB) and mitogen-activated protein kinase (MAPK) [77,81]. Finally, in 3T3-L1 cells, 1,25(OH)2D at physiological doses inhibited apoptosis, and at high doses stimulated it [82]. Apoptosis is induced and mediated by calcium, and 1,25(OH)2D, at high doses, activates calcium-dependent apoptotic proteases in adipose tissue [83].

The present study employs various methods for assessing body composition, aiming to elucidate its relationship with serum vitamin D concentrations, particularly in women of childbearing age. This constitutes a strong point, allowing for a more comprehensive understanding of the interplay between vitamin D, body adiposity, and muscle mass. However, it is important to acknowledge some limitations of this research, such as the cross-sectional model, which restricts the ability to establish temporal relationships or observe changes over time. Additionally, the absence of sun exposure assessment and the inability to generalize the results to other demographic groups beyond the specified age range are aspects to be considered.

## 5. Conclusions

We observed a high percentage of vitamin D status inadequacy related to lower muscle mass and higher body fat (total and visceral). We also observed a positive correlation between 25(OH)D and muscle mass and a negative correlation with body fat. These findings suggest that inadequate levels of this vitamin may affect muscle tissue and be involved with higher body adiposity, including visceral adiposity.

We suggest the inclusion of these body variables in clinical practice, especially WHtR and SMI _BMI_, to minimize the metabolic consequences resulting from vitamin inadequacy.

## Figures and Tables

**Table 1:** Indices and formulas for estimating body fat.

Index	Formula	Reference
Body mass index (BMI)	BMI (kg/m^2^) = $\frac{\mathrm{weight}\;\left(\mathrm{kg}\right)}{\mathrm{height}\;\left(m\right)}$	WHO, 2000 [26]
Waist-to-hip ratio (WHR)	$\mathrm{WHR}=\frac{\mathrm{WC}\;\left(\mathrm{cm}\right)}{\mathrm{HC}\;\left(\mathrm{cm}\right)}$	WHO, 2008 [30]
Waist-to-height ratio (WHtR)	$\mathrm{WHtR}=\frac{\mathrm{WC}\;\left(\mathrm{cm}\right)}{\mathrm{height}\;\left(\mathrm{cm}\right)}$	Ashwell; Lejeune; Mcpherson, 1996 [32]
Conicity index (CI)	CI = $\frac{\mathrm{WC}\;\left(m\right)}{0.109\times \sqrt{\mathrm{weight}\;\left(\mathrm{kg}\right)/\mathrm{height}\;\left(m\right)}}$	Valdez, 1991 [31]
Body adiposity index (BAI)	BAI (%) = $\frac{\mathrm{HC}\;\left(\mathrm{cm}\right)}{\mathrm{height}\;{\left(m\right)}^{1.5}}-18$	Bergman et al., 2011 [34]
A body shape index (ABSI)	ABSI (m^11/6^ kg^−2/3^) = $\frac{\mathrm{WC}\;\left(m\right)}{\mathrm{BMI}\;{\left(\mathrm{kg}/m^{2}\right)}^{2/3}\times \;\mathrm{height}{\left(m\right)}^{1/2}}$	Krakauer; Krakauer, 2012 [35]
Clínica Universidad de Navarra—Body Adiposity Estimator (CUN-BAE)	CUN-BAE (%) = −44.988 + (0.503 × age (years)) + (10.689 × sex) + (3172 × BMI (kg/m^2^)) − (0.026 × BMI (kg/m^2^) ^2^) + (0.181 × BMI (kg/m^2^) × sex) − (0.02 × BMI (kg/m^2^) × age (years)) − (0.005 × BMI (kg/m^2^) ^2^ × sex) + (0.00021 × BMI (kg/m^2^) ^2^ × age (years)) Sex: female = 1	Gómez-Ambrosi et al., 2012 [36]
Belarmino–Waitzberg index (BeW)	Woman BeW (%) = −48.8 + 0.087 × WC (cm) + 1.147 × HC (cm) − 0.003 × HC (cm) ^2^	Belarmino et al., 2018 [37]
Visceral adiposity index (VAI)	$\mathrm{VAI}\;(\mathrm{women})=\left(\frac{\mathrm{WC}\;\left(\mathrm{cm}\right)}{39.58+\left(1.89\times \;\mathrm{IMC}\;\left(\mathrm{kg}/m^{2}\right)\right)}\right)$ $\;\times \;\left(\frac{\mathrm{TG}\;\left(\mathrm{mmol}/L\right)}{0.81}\right)$ $\;\times \;\left(\frac{1.52}{\mathrm{HDL}\;\left(\mathrm{mmol}/l\right)}\right)$	Amato et al., 2010 [33]
Fat mass index (FMI)	FMI (kg/m^2^) = $\frac{\mathrm{FM}\;\left(\mathrm{kg}\right)}{\mathrm{height}\;(m{)}^{2}}$	Schutz; Kyle; Pichard, 2002 [38]
Fat-to-muscle ratio (FMR)	$\mathrm{FMR}=\frac{\mathrm{FM}\;\left(\mathrm{kg}\right)}{\mathrm{MM}\;\left(\mathrm{kg}\right)}$	Xu et al., 2018 [39]

**Table 2:** Indices and formulas for estimating muscle mass.

Index	Formula	Reference
Arm muscle circumference (AMC)	AMC (cm) = AC (cm) − (DCT (mm) × 0.3142)	Frisancho, 1974 [28]
Arm muscle area (AMA)	AMA (cm^2^) = $\frac{{\left(\mathrm{AC}\;\left(\mathrm{cm}\right)-\left[\mathrm{DCT}\;\left(\mathrm{mm}\right)\times 0.3142\right]\right)}^{2}}{12.57}$	Gurney; Jelliffe, 1973 [29]
Fat-free mass index (FFMI)	$\mathrm{FFMI}\;(\mathrm{kg}/m^{2})=\frac{\mathrm{FFM}\;\left(\mathrm{kg}\right)}{\mathrm{height}\;(m{)}^{2}}$	Schutz; Kyle; Pichard, 2002 [38]
Muscle mass index adjusted by height^2^ (SMI _height_)	$\mathrm{SMI}{\;}_{\mathrm{height}}\;(\mathrm{kg}/m^{2})=\frac{\mathrm{SMM}\;\left(\mathrm{kg}\right)}{\mathrm{height}\;(m{)}^{2}}$	Baumgartner et al., 1998 [40]
Muscle mass index adjusted by weight (SMI _weight_)	$\mathrm{SMI}{\;}_{\mathrm{weight}}=\frac{\mathrm{SMM}\;\left(\mathrm{kg}\right)}{\mathrm{weight}\;\left(\mathrm{kg}\right)}\times 100$	Janssen; Heymsfield; Ross, 2002 [41]
Muscle mass index adjusted by BMI (SMI _BMI_)	$\mathrm{SMI}{\;}_{\mathrm{BMI}}=\frac{\mathrm{SMM}\;\left(\mathrm{kg}\right)}{\mathrm{BMI}\;\left(\mathrm{kg}/m^{2}\right)}$	Cawthon et al., 2014 [42]

**Table 3:** General characteristics of the sample comprising 124 adult women of childbearing age, classified in accordance with Vitamin D status into sufficient, insufficient, deficient, adequate, and inadequate (mean ± sd).

	Vitamin D (25(OH)D)				Vitamin D (25(OH)D)		
	Sufficient (n = 45)	Insufficient (n = 55)	Deficient (n = 24)	*p* ^a^	Adequate (n = 45)	Inadequate (n = 79)	*p*
25(OH)D (ng/mL)	40.07 ± 8.24 ^b,c^	24.56 ± 2.68 ^b,d^	17.55 ± 1.60 ^c,d^	<0.001	40.07 ± 8.24	22.43 ± 4.03	<0.001
Age (years)	33.78 ± 7.54	33.33 ± 6.50	36.33 ± 7.55	0.200	33.78 ± 7.54	34.24 ± 6.93	0.660
Weight (kg)	63.07 ± 7.87	66.62 ± 13.47	65.36 ± 9.87	0.496	63.07 ± 7.87	66.24 ± 12.44	0.239
WC (cm)	78.83 ± 7.02	81.89 ± 10.49	82.46 ± 8.88	0.249	78.83 ± 7.02	82.06 ± 9.97	0.114
HC (cm)	99.63 ± 6.14	102.67 ± 9.45	101.52 ± 8.08	0.382	99.63 ± 6.14	102.31 ± 9.01	0.181
AC (cm)	27.57 ± 2.43	27.32 ± 3.05	27.98 ± 2.67	0.607	27.56 ± 2.43	27.54 ± 2.93	0.900
TSF (mm)	26.75 ± 6.71	27.45 ± 7.67	27.68 ± 6.99	0.918	26.75 ± 6.71	27.53 ± 7.39	0.680
AMC (cm)	19.16 ± 1.22	18.70 ± 1.80	19.29 ± 1.48	0.166	19.16 ± 1.22	18.89 ± 1.71	0.256
AMA (cm^2^)	29.34 ± 3.78	28.09 ± 5.54	29.78 ± 4.62	0.166	29.34 ± 3.78	28.65 ± 5.28	0.256
BMI (kg/m^2^)	23.45 ± 2.85	24.80 ± 4.02	24.24 ± 3.03	0.284	23.44 ± 2.85	24.63 ± 3.73	0.115
WHR	0.79 ± 0.05	0.80 ± 0.06	0.81 ± 0.06	0.307	0.79 ± 0.05	0.80 ± 0.06	0.302
CI	1.17 ± 0.06	1.18 ± 0.07	1.20 ± 0.08	0.144	1.17 ± 0.58	1.19 ± 0.73	0.116
WHtR	0.48 ± 0.05 ^b,c^	0.50 ± 0.06 ^b^	0.50 ± 0.05 ^c^	0.062	0.48 ± 0.05	0.50 ± 0.05	0.021
VAI	1.26 ± 0.88	1.00 ± 0.46	1.50 ± 1.12	0.291	1.26 ± 0.88	1.14 ± 0.74	0.688
BAI (%)	29.49 ± 3.44 ^b^	31.24 ± 3.86 ^b^	30.26 ± 3.60	0.034	29.49 ± 3.44	30.97 ± 3.78	0.019
ABSI (m^11/6^ kg^−2/3^)	0.12 ± 0.01	0.12 ± 0.01	0.13 ± 0.01	0.434	0.12 ± 0.01	0.12 ± 0.01	0.514
CUN-BAE (%)	32.12 ± 4.36	33.95 ± 5.79	33.68 ± 4.68	0.192	32.12 ± 4.36	33.87 ± 5.45	0.074
BeW (%)	42.45 ± 3.80	44.21 ± 5.72	43.71 ± 4.88	0.378	42.45 ± 3.80	44.06 ± 5.45	0.166
BF (kg)	19.51 ± 6.13	22.31 ± 9.36	22.21 ± 6.59	0.336	19.51 ± 6.13	22.29 ± 8.58	0.202
FFM (kg)	42.98 ± 4.31	43.12 ± 6.07	42.66 ± 4.73	0.984	42.98 ± 4.31	42.99 ± 5.67	0.958
SMM (kg)	23.45 ± 2.55	23.30 ± 4.14	23.16 ± 2.77	0.974	23.45 ± 2.55	23.25 ± 3.77	0.890
% BF (%)	30.73 ± 6.37	33.00 ± 7.53	33.69 ± 6.51	0.203	30.73 ± 6.37	33.20 ± 7.20	0.094
FMI (kg/m^2^)	7.27 ± 2.34	8.33 ± 3.24	8.21 ± 2.45	0.270	7.27 ± 2.34	8.39 ± 3.01	0.132
FFMI (kg/m^2^)	15.98 ± 1.25	16.17 ± 1.47	15.75 ± 1.16	0.511	15.98 ± 1.25	16.04 ± 1.39	0.692
FMR	0.84 ± 0.26	0.95 ± 0.32	0.96 ± 0.28	0.166	0.84 ± 0.26	0.95 ± 0.30	0.065
SMI _height_ (kg/m^2^)	8.72 ± 0.76	8.73 ± 1.20	8.55 ± 0.71	0.522	8.72 ± 0.76	8.68 ± 1.08	0.846
SMI _weight_ (%)	37.78 ± 3.56	36.02 ± 4.69	35.97 ± 3.69	0.141	37.78 ± 3.56	36.01 ± 4.39	0.051
SMI _BMI_	1.02 ± 0.13 ^b^	0.96 ± 0.15 ^b^	0.97 ± 0.12	0.106	1.02 ± 0.13	0.96 ± 0.14	0.039

Sufficient vitamin D (25(OH)D ≥ 30 ng/mL); Insufficient vitamin D (25(OH)D ≥ 20 and <30 ng/mL); Deficient vitamin D (25(OH)D < 20 ng/mL); adequate vitamin D (25(OH)D ≥ 30 ng/mL); inadequate vitamin D (25(OH)D < 30 ng/mL). WC: Waist circumference; HC: Hip circumference; AC: Arm circumference; TSF: Triceps skinfold; AMC: Arm muscle circumference; AMA: Arm muscle area; BMI: Body mass index; WHR: Waist-to-hip ratio; CI: Conicity index; WHtR: Waist-to-height ratio; VAI: Visceral adiposity index; BAI: Body adiposity index; ABSI: A body shape index; CUN-BAE: Clínica Universidad de Navarra—Body adiposity estimator; BeW: Belarmino–Waitzberg index; FM: Fat mass; FFM: Fat-free mass; SMM: Skeletal muscle mass; % BF: Body fat percentage; FMI: Fat mass index; FFMI: Fat-free mass index; FMR: Fat–muscle ratio; SMI _height_: Height-adjusted muscle mass index^2^; SMI _weight_: Weight-adjusted muscle mass index; SMI _BMI_: BMI-adjusted muscle mass index. ^a^ Comparisons among the three groups analyzed by the Kruskal–Wallis test. ^b^ Separate post-test (Mann–Whitney) showed (*p* < 0.05) between sufficient and insufficient. ^c^ Separate post-test (Mann-Whitney) showed (*p* < 0.05) between sufficient and deficient. ^d^ Separate post-test (Mann-Whitney) showed (*p* < 0.05) between insufficient and deficient.

**Table 4:** General characteristics of the sample comprising 124 adult women of childbearing age, classified in accordance with their Vitamin D status as adequate or inadequate and BMI (mean ± sd).

	Adequate Vitamin D		Inadequate Vitamin D		*p* *
	BMI Normal Weight (n = 35)	BMI Overweight (n = 10)	BMI Normal Weight (n = 48)	BMI Overweight (n = 31)	
25(OH)D (ng/mL)	40.89 ± 8.12 ^c,e^	37.17 ± 8.41 ^d,f^	22.66 ± 4.16 ^c,f^	22.08 ± 3.87 ^d,e^	<0.001
Age (years)	33.14 ± 7.52	36.00 ± 7.59	34.63 ± 6.62	33.65 ± 7.45	0.619
Weight (kg)	60.79 ± 6.52 ^a,e^	71.03 ± 7.18 ^a,d,f^	58.60 ± 7.27 ^b,f^	78.07 ± 9.01 ^b,d,e^	<0.001
WC (cm)	76.03 ± 4.96 ^a,e^	88.65 ± 3.16 ^a,f^	76.33 ± 6.20 ^b,f^	90.94 ± 8.03 ^b,e^	<0.001
HC (cm)	97.77 ± 5.20 ^a,e^	106.15 ± 4.66 ^a,d,f^	96.59 ± 5.24 ^b,f^	111.00 ± 6.08 ^b,d,e^	<0.001
AC (cm)	26.79 ± 1.92 ^a,e^	30.56 ± 1.83 ^a,f^	26.23 ± 2.17 ^b,f^	30.76 ± 1.86 ^b,e^	<0.001
TSF (mm)	25.34 ± 6.37 ^a,e^	32.22 ± 5.24 ^a,f^	24.62 ± 6.02 ^b,f^	34.74 ± 5.27 ^b,e^	<0.001
AMC (cm)	18.83 ± 1.01 ^a,e^	20.44 ± 1.18 ^a,f^	18.50 ± 1.62 ^b,f^	19.86 ± 1.59 ^b,e^	<0.001
AMA (cm^2^)	28.30 ± 3.02 ^a,e^	33.35 ± 3.88 ^a,f^	27.47 ± 4.95 ^b,f^	31.58 ± 5.03 ^b,e^	<0.001
BMI (kg/m^2^)	22.20 ± 1.37 ^a,e^	27.80 ± 2.36 ^a,f^	22.15 ± 1.59 ^b,f^	28.46 ± 2.70 ^b,e^	<0.001
WHR	0.78 ± 0.05 ^a,e^	0.84 ± 0.04 ^a,f^	0.79 ± 0.05 ^b^	0.82 ± 0.06 ^b,e^	0.001
CI	1.15 ± 0.05 ^a,e^	1.22 ± 0.05 ^a,f^	1.17 ± 0.06 ^b,f^	1.22 ± 0.08 ^b,e^	<0.001
WHtR	0.46 ± 0.03 ^a,c,e^	0.56 ± 0.03 ^a,f^	0.47 ± 0.03 ^b,c,f^	0.55 ± 0.05 ^b,e^	<0.001
VAI	1.08 ± 0.55	1.83 ± 1.41	1.02 ± 0.61	1.30 ± 0.86	0.142
BAI (%)	28.04 ± 1.95 ^a,e^	34.58 ± 2.55 ^a,f^	28.86 ± 2.44 ^b,f^	34.17 ± 3.17 ^b,e^	<0.001
ABSI (m^11/6^kg^−2/3^)	0.12 ± 0.01	0.12 ± 0.01	0.12 ± 0.01	0.13 ± 0.01	0.530
CUN-BAE (%)	30.23 ± 2.27 ^a,e^	38.75 ± 3.30 ^a,f^	30.33 ± 2.93 ^b,f^	39.34 ± 3.59 ^b,e^	<0.001
BeW (%)	41.20 ± 3.15 ^a,e^	46.80 ± 2.44 ^a,d,f^	40.56 ± 3.34 ^b,f^	49.36 ± 3.29 ^b,d,e^	<0.001
FM (kg)	1768 ± 4.41 ^a,e^	27.10 ± 6.73 ^a,f^	17.33 ± 4.23 ^b,f^	31.72 ± 6.59 ^b,e^	<0.001
AMC (kg)	42.74 ± 4.55 ^e^	43.96 ± 3.18	41.33 ± 5.37 ^b^	46.15 ± 4.92 ^b,e^	0.005
SMM (kg)	23.28 ± 2.68 ^e^	24.16 ± 1.91	22.14 ± 3.69^b^	25.38 ± 2.96 ^b,e^	0.003
% BF (%)	29.02 ± 5.39 ^a,e^	37.77 ± 5.40 ^a,f^	29.39 ± 5.18 ^b,f^	40.47 ± 4.32 ^b,e^	<0.001
FMI (kg/m^2^)	6.48 ± 1.49 ^a,e^	10.55 ± 2.44 ^a,f^	6.53 ± 1.40 ^b,f^	11.66 ± 2.28 ^b,e^	<0.001
FFMI (kg/m^2^)	15.70 ± 1.12 ^a,e^	17.15 ± 1.15 ^a,f^	15.58 ± 1.25 ^b,f^	16.94 ± 1.21 ^b,e^	<0.001
FMR	0.77 ± 0.21 ^a,e^	1.12 ± 0.25 ^a,f^	0.80 ± 0.20 ^b,f^	1.25 ± 0.23 ^b,e^	<0.001
SMI _height_ (kg/m^2^)	8.55 ± 0.68 ^a,e^	9.42 ± 0.72 ^a,f^	8.35 ± 1.08 ^b,f^	9.31 ± 0.77 ^b,e^	<0.001
SMI _weight_ (%)	38.64 ± 3.13 ^a,e^	34.19 ± 3.06 ^a,f^	37.74 ± 4.22 ^b,f^	32.71 ± 2.40 ^b,e^	<0.001
SMI _BMI_	1.05 ± 0.11 ^a,e^	0.88 ± 0.08 ^a,f^	1.00 ± 0.15 ^b,f^	0.89 ± 0.09 ^b,e^	<0.001

Adequate vitamin D (25(OH)D ≥ 30 ng/mL); Inadequate vitamin D (25(OH)D < 30 ng/mL); Normal weight BMI (BMI ≥ 18.5 and ≤ 24.9 kg/m^2^); Overweight BMI (BMI ≥ 25.0 kg/m^2^). 25(OH)D: 25 hydroxy vitamin D; WC: Waist circumference; HC: Hip circumference; AC: Arm circumference; TSF: Triceps skinfold; AMC: Arm muscle circumference; AMA: Arm muscle area; BMI: Body mass index; WHR: Waist-to-hip ratio; CI: Conicity index; WtHR: Waist-to-height ratio; VAI: Visceral adiposity index; BAI: Body adiposity index; ABSI: Body shape index; CUN-BAE: Clínica Universidad de Navarra—Body adiposity estimator; BeW: Belarmino–Waitzberg index; FM: Fat mass; FFM: Fat-free mass; SMM: Skeletal muscle mass; BF %: Percent Body fat percent; FMI: Fat mass index; FFMI: Fat-free mass index; FMR: Fat–muscle ratio; SMI _height_: Height-adjusted muscle mass index^2^; SMI _weight_: Weight-adjusted muscle mass index; SMI _BMI_: BMI-adjusted muscle mass index. * Comparisons between the four groups analyzed by the Kruskal–Wallis test. ^a^ Separate post-test (Mann–Whitney) showed (*p* < 0.05) between adequate Vitamin D with normal-weight BMI and adequate Vitamin D with overweight BMI. ^b^ Separate post-test (Mann–Whitney) showed (*p* < 0.05) between inadequate Vitamin D with normal-weight BMI and inadequate Vitamin D with overweight BMI. ^c^ Separate post-test (Mann–Whitney) showed (*p* < 0.05) between adequate Vitamin D with normal-weight BMI and inadequate Vitamin D with normal-weight BMI. ^d^ Separate post-test (Mann–Whitney) showed (*p* < 0.05) between adequate Vitamin D with overweight BMI and inadequate Vitamin D with overweight BMI. ^e^ Separate post-test (Mann–Whitney) showed (*p* < 0.05) between adequate Vitamin D with normal-weight BMI and inadequate Vitamin D with overweight BMI. ^f^ Separate Post-test (Mann–Whitney) showed (*p* < 0.05) between adequate Vitamin D with overweight BMI and inadequate Vitamin D with normal-weight BMI.

**Table 5:** Correlations between circulating concentrations of 25(OH)D, age, and body variables of 124 adult women of childbearing age.

	Correlation (r)	*p*
Age (years)	−0.068	0.451
Weight (kg)	−0.111	0.221
WC (cm)	−0.194	0.031
HC (cm)	−0.123	0.174
AC (cm)	−0.073	0.451
TSF (mm)	−0.132	0.170
AMC (cm)	0.063	0.514
AMA (cm^2^)	0.050	0.605
BMI (kg/m^2^)	−0.151	0.094
WHR	−0.175	0.053
CI	−0.207	0.021
WHtR	−0.218	0.015
VAI	−0.024	0.802
BAI (%)	−0.172	0.057
ABSI (m^11/6^ kg^−2/3^)	−0.093	0.304
CUN-BAE (%)	−0.161	0.073
BeW (%)	−0.132	0.145
FM (kg)	−0.189	0.063
FFM (kg)	0.050	0.627
SMM (kg)	−0.044	0.666
% BF (%)	−0.214	0.035
FMI (kg/m^2^)	−0.210	0.039
FFMI (kg/m^2^)	−0.008	0.940
FMR	−0.226	0.026
SMI _height_ (kg/m^2^)	−0.003	0.980
SMI _weight_ (%)	0.199	0.051
SMI _BMI_	0.219	0.031

WC: Waist circumference; HC: Hip circumference; AC: Arm circumference; TSF: Triceps skinfold; AMC: Arm muscle circumference; AMA: Arm muscle area; BMI: Body mass index; WHR: Waist-to-hip ratio; CI: Conicity index; WHtR: Waist-to-height ratio; VAI: Visceral adiposity index; BAI: Body adiposity index; ABSI: Body shape index; CUN-BAE: Clínica Universidad de Navarra—Body adiposity estimator; BeW: Belarmino–Waitzberg index; FM: Fat mass; FFM: Fat-free mass; SMM: Skeletal muscle mass; % BF: Percent body fat; FMI: Fat mass index; FFMI: Fat-free mass index; FMR: Fat–muscle ratio; SMI _height_: Height-adjusted muscle mass index^2^; SMI _weight_: Weight-adjusted muscle mass index; SMI _BMI_: BMI-adjusted muscle mass index.

## Data Availability

The data used to support the findings of this study are available from the corresponding author upon request. Data is not publicly available due to privacy.
